# Annotations

**Published:** 1906-04-14

**Authors:** 


### ANNOTATIONS.

### Sudden Deaths and Medical Reputations.

Mr. Alfred Parkin has recently drawn attention to the embarrassment in which a medical man may find himself lodged by the sudden death of a patient held to be convalescent. The case in point was that of a lady who fell ill with sciatica. She was persuaded to go to bed, and progressed well for some days. An attack of bronchitis complicated by pleurisy confined her to her bed for some time longer, but at last she appeared to be definitely on the mend. At this period, there being no reason to anticipate anything untoward, she became suddenly worse. Mr. Parkin hurried to her side to find her moribund, and she died within a few minutes of his arrival. In point of fact, as was shown by autopsy, she had died of pulmonary embolism. It is easy to reconstruct the train of thought likely to present itself under such circumstances to an uninstructed and distressed relative of the patient. He would argue that an illness, so grave as to be liable to a fatal termination at a moment's notice, must present signals of danger to the seeing eye. Therefore the doctor has not the seeing eye. But he is paid for having it; therefore he is inefficient, if not actually an undeserving rogue. What explanation is to be given by the doctor? Mr. Parkin advises what certainly seems the correct course—namely, to enumerate the possible explanations, to affirm the fact that there was no reason to anticipate any one of them, and to advise a post-mortem examination; but the strength of this position depends, of course, upon care in the clinical examination of the case during life. Such difficulties as these are certain to present themselves from time to time, and must continue to be disconcerting so long as the public remains in ignorance of the intrinsically small mechanical alterations which suffice to throw the circulatory apparatus suddenly out of gear. This will probably be for ever, for it is not to be supposed that people will cease to blame the doctor for not detecting an ante-mortem clot in the chambers of the heart. We must endeavour to console ourselves with the implied compliment to our professional abilities.

### Typhoid Bacilluria.

Dr. Dods Brown, in the "Edinburgh Medical Journal" for February, draws attention to a feature of typhoid fever that possibly is not sufficiently widely recognised—the presence of typhoid bacilli in the urine. Many observers have noted their presence in typhoid fever, but Dr. Brown considers that only thirteen papers upon the subject fall within the "period of reliable bacteriology." The experience of different observers has not been precisely similar, but the results of investigations given in eleven of these thirteen papers give the percentage of frequency of typhoid bacilli in the urine in typhoid fever at from 18 to 42 per cent. Dr. Dods Brown examined the urine in fifteen cases, and found the bacillus in eight—that is, in just over half the cases. The time of appearance of the bacilli seems to have varied considerably, but it has been most commonly in the third or fourth week. When once they have appeared, however, they may persist for several weeks, or even months. The number present is said sometimes to be very great. Gwyn is quoted as having estimated that 500,000 million would probably be present in the urine passed during twenty-four hours. It is scarcely necessary to remark that the presence of these bacilli in the urine increases the risk of infection in others. The urine should therefore be treated with disinfectants. It is also necessary to remember that dried urine on bed-clothes may be a source of danger. Urotropine taken internally causes the bacilli to disappear from the urine of the patient, and it may be advisable to give urotropine for two or three days at a time to typhoid patients as a routine measure.

### The Treatment of Sarcoma by Mixed Toxins.

It is interesting to gain further information from Dr. Coley of the results of the treatment of inoperable sarcoma by the mixed toxins of erysipelas and bacillus prodigiosus. Many medical men in this country, no doubt, have tried this treatment with disappointing results. While, however, there cannot but have been many instances of failure, it is interesting to note that Dr. Coley is able to tabulate sixty cases under the care of various surgeons where the employment of these toxins has resulted in complete or partial cure. Amongst these surgeons who have had satisfactory results are several attached to the staffs of hospitals in London. In addition to a summary of these sixty cases treated by others, Dr. Coley gives details of thirty-six cases under his own care in which there had been varying degrees of success. Of these thirty-six cases twenty-one remained well from periods varying between five and thirteen years. In five cases in which the tumour temporarily disappeared recurrence occurred and caused the death of the patients. Of these five cases Dr. Coley speaks as follows. He says they are "perhaps the most important of all, inasmuch as they furnish absolute proof of the correctness of the diagnosis and refute the early objections brought against the method that the successful cases were probably examples of errors in the diagnosis." Dr. Coley also lays stress upon the fact that it must be remembered that not only were his cases proved by microscopical examination to be sarcoma, but that they were cases which, from the operative point of view, were "absolutely hopeless." To hear of a tumour the size of a child's head disappearing under this treatment is, it is needless to say, remarkable, and introduces at least a ray of hope in dealing with some cases before which generally nothing can be felt by the medical attendant but complete powerlessness. In considering these cases it must be remembered that instances of malignant growth are in very rare instances met with which, even when they may have reduced a patient almost to a skeleton, the growth loses its vitality, ceases to increase in size, and then gradually atrophies and disappears, while the patient gains flesh and returns, it may be, to perfect health. Such cases are, however, far too rare to explain the successful results of Dr. Coley, but they seem to show that the human body under certain circumstances has power to inhibit the growth of, and finally cause to disappear, a malignant tumour by which it has become attacked.

